# Tetramerization of upstream stimulating factor USF2 requires the elongated bent leucine zipper of the bHLH-LZ domain

**DOI:** 10.1016/j.jbc.2023.105240

**Published:** 2023-09-09

**Authors:** Cao Huang, Mingyu Xia, Hang Qiao, Zaizhou Liu, Yuqi Lin, Hanyin Sun, Biao Yu, Pengfei Fang, Jing Wang

**Affiliations:** 1 School of Materials and Chemistry, University of Shanghai for Science and Technology, Shanghai, China; 2 State Key Laboratory of Chemical Biology, Shanghai Institute of Organic Chemistry, Chinese Academy of Sciences, Shanghai, China; 3 School of Chemistry and Materials Science, Hangzhou Institute for Advanced Study, University of Chinese Academy of Sciences, Hangzhou, China

**Keywords:** transcription factor, upstream stimulating factor (USF), basic Helix-Loop-Helix Leucine Zipper (bHLH-LZ) domain, DNA complex structure, DNA looping

## Abstract

Upstream stimulating factors (USFs), including USF1 and USF2, are key components of the transcription machinery that recruit coactivators and histone-modifying enzymes. Using the classic basic helix-loop-helix leucine zipper (bHLH-LZ) domain, USFs bind the E-box DNA and form tetramers that promote DNA looping for transcription initiation. The structural basis by which USFs tetramerize and bind DNA, however, remains unknown. Here, we report the crystal structure of the complete bHLH-LZ domain of USF2 in complex with E-box DNA. We observed that the leucine zipper (LZ) of USF2 is longer than that of other bHLH-LZ family transcription factors and that the C-terminus of USF2 forms an additional α-helix following the LZ region (denoted as LZ-Ext). We also found the elongated LZ-Ext facilitates compact tetramer formation. In addition to the classic interactions between the basic region and DNA, we show a highly conserved basic residue in the loop region, Lys271, participates in DNA interaction. Together, these findings suggest that USF2 forms a tetramer structure with a bent elongated LZ-Ext region, providing a molecular basis for its role as a key component of the transcription machinery.

Upstream stimulating factors (USFs) were first identified in HeLa cell nuclear extracts to bind a palindromic DNA sequence (GGCCACGTGACC) in the adenovirus major late promoter and result in a 10 to 20-fold increase in transcription (1). Further studies showed that USFs, including USF1 and USF2, are ubiquitously expressed in eukaryotes and interact with cognate E-box regulatory elements (CANNTG) throughout the genome with high affinity. USFs directly interact with the transcription factor II D and TATA-binding protein-associated factors for TATA-directed gene transcription (1, 2, 3, 4), and other transcription factors, including specificity protein 1, polyomavirus enhancer activator 3, and metal-responsive transcription factor 1 (5, 6, 7). In addition, USFs mediate the recruitment of histone-modifying enzymes, such as the acetyltransferase P300/CBP-associated factor and the methyltransferase SET domain-containing protein 7 (SET7/9) (8). With extensive and hierarchical roles in transcriptional regulation, USFs are considered to be key components of the transcription machinery (9). They play crucial roles in stress and immune responses, glucose responses, mitochondrial homeostasis, and cell growth and development (10, 11, 12, 13).

USFs belong to the basic helix-loop-helix leucine zipper (bHLH-LZ) transcription factor family, which also includes the MiT/TFE members microphthalmia-associated transcription factor (MITF), transcription factor EB (TFEB), transcription factor EC, and transcription factor E3 (TFE3) and the MYC-subfamily members myc proto-oncogene (MYC), MYC-associated factor X (MAX), MAX dimerization protein, and MAX interacting protein 1 (MXI1) (9). The bHLH-LZ domain is a classic DNA-binding module of which helix-loop-helix and leucine zipper (LZ) regions mediate the formation of homodimers or heterodimers, forming scaffolds to support the appropriate position of the basic region, which directly interacts with the major groove of E-box DNA (14). The LZ region also participates in the recruitment of transcription initiation coactivators. For example, the LZ region of USF1 is required to form a USF1/polyomavirus enhancer activator 3/DNA ternary complex for the cooperative transcription of the *Bax* gene (15).

Tetramerization has been found to be important for the functioning of some transcription factors such as p53 (16), developmental protein SEPALLATA 3 (17), and signal transducer and activator of transcription 5 (18). USFs also form a bivalent homotetramer in solution. The tetramer could interact with two E-box DNA recognition sites simultaneously and is predicted to mediate USF-looped DNA complex formation for transcriptional activation (19). Biochemical analysis showed that the integrity of the LZ region of USF was required for tetramer formation (20). However, the structural basis of USF tetramerization remains unknown.

USF1 and USF2 share 44% sequence identity and redundant functions (21, 22), and they also have some functional differences. For example, USF2 may play a more dominant role than USF1 in the process of embryonic and adult development (23, 24). Here, we report the crystal structure of the complete bHLH-LZ domain of USF2 in complex with E-box DNA. We observed that the rest of the C terminus of USF2 forms an additional α-helix following the LZ region. The elongated LZ significantly deviated from those of other bHLH-LZ family transcription factors. Significantly, two USF2 dimers form a compact tetramer that can support USF-looped DNA complex formation. In addition to the classic interactions between the basic region and DNA, we observed a conserved Lys271 in the loop region that contributes to DNA recognition. Together, these results provide the structural basis to understand the role of USF2 as one of the key components of the transcription machinery.

## Results

### Structure of the bHLH-LZ-Ext domain of human USF2 in complex with E-box DNA

**Figure 1:**
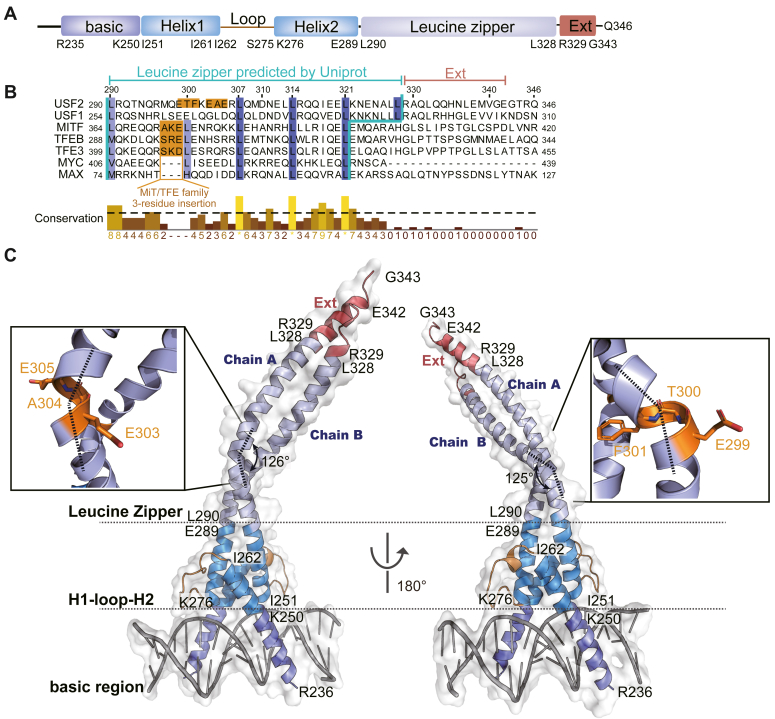
**The overall complex structure of USF2 and E-box DNA.***A*, schematic cartoon of the USF2 bHLH-LZ domain and the Ext region. *B*, sequence alignment between USFs, MiT/TFE family transcription factors and MYC/MAX transcription factors. *C*, overall structure of USF2 bHLH-LZ-Ext complexed with the E-box DNA. The bending points of USF2 _303_EAE_305_ in chain A and _299_ETF_301_ in chain B are highlighted in *orange*. Color coding: basic region (Arg236–Lys250): *purple*; Helix1 and Helix2 region (Ile251–Ile261, Lys276–Glu289): *blue*; Loop region (Ile262–Ser275): *yellow*; Leucine zipper region (Leu290–Leu328): *light purple*; Ext (Arg329–Gly343): *red* and E-box DNA: *gray*. MAX, MYC-associated factor X; USF, upstream stimulating factor.

USFs have a unique bHLH-LZ domain, especially its LZ region (Fig. 1*A*). Previous research by us and other groups found that there is a “3-residue insertion” in the LZ region of MiT/TFE members but not MYC subfamily members that interferes with the heptad leucine repeat (Fig. 1*B*) (25, 26). This insertion in MITF makes its LZ highly dynamic, and its dimer unstable (25). USF2 LZ region is even more interesting. It also contains a “3-residue insertion” in the LZ region, and the conserved leucine residue next to the insertion in the MiT/TFE family is missing in USF2 (Fig. 1*B*). In addition, USFs have one additional set of seven residues spaced by leucine (from Leu321 to Leu328 in USF2) compared with other bHLH-LZ transcription factors (Fig. 1*B*). It is intriguing to know what structural and functional properties USF2 has based on these unique sequence features.

To investigate the structure of USF2, we prepared a recombinant protein fragment of human USF2 from the basic region to the C terminus (isoform 1, residues 235–346), including the complete bHLH-LZ domain according to the UniProt database annotation (Fig. 1, *A* and *B*). We cocrystallized this protein with E-box DNA and solved the crystal structure to a resolution of 3.5 Å (Fig. 1*C* and Table 1). The crystal belonged to the orthorhombic system space group ***I***121. One asymmetric unit (ASU) contains four protein chains (A, B, E, and F) and four single strands of DNA (chains C, D, G, and H) (Fig. S1*A*). Chains A and B as well as E and F form compact dimers to bind duplex E-box DNAs formed in pairs of C and D chains and G and H chains, respectively. Therefore, there are two dimeric USF2-DNA complexes in an ASU. The structures of the two dimers closely resembled each other with a RMSD of 0.36 Å (Fig. S1*B*).

**Table 1:** Data collection and refinement statistics

	USF2_bHLH-LZ-ext
Data collection	
Space group	*I 1 2 1*
Cell dimensions	
*a*, *b*, *c* (Å)	78.85, 56.95, 232.12
α, β, γ (°)	90.00, 90.92, 90.00
Resolution (Å)	37.14–3.50 (3.63–3.50)
*R*_sym_ or *R*_merge_ (%)	15.1 (70.8)
Mean I/sigma (I)	5.2 (1.6)
Completeness (%)	98.31 (97.36)
Redundancy	1.8 (1.9)
Refinement	
Total reflections	12,998 (1246)
*R*_work_/*R*_free_ (%)	24.33/29.59
No. atoms	4613
Average B-factors	104.57
R.m.s. deviations	
Bond length (Å)	0.004
Bond angle (°)	0.737
Ramachandran plot	
*Most favored* [%]	98.55
*Additional allowed* [%]	1.45

In each dimeric USF2-DNA complex, the basic region (from Arg236 to Lys250) forms a helix to bind the major groove of E-box DNA (Fig. 1*C*). Helices 1 and 2 of both chains form a hydrophobic four-helix bundle through interactions between residues Ile251, Ile255, Leu258, Ile261, Leu280, Tyr286, and Arg291 (Fig. S2). The complete LZ region from Leu290 to Leu328 was resolved in the structure. Interestingly, the rest of the C terminus of one chain of the dimer from Arg329 to Glu342 also forms an α-helix extending LZ (hereafter denoted as the Ext region) (Fig. 1*C*).

LZ-Ext adopts a prominent bent structure with helixes twisted by approximately 125° in both chain A and chain B (Fig. 1*C*). The bending of LZ caused by the “3-residue insertion” has been revealed in the crystal structures of MITF (25), TFEB (27), and TFE3 (28). In these MiT/TFE members, the turning points are all located on the “3-residue insertion”. Because USF2 lacks one regular leucine in the LZ region, it is difficult to distinguish which three residues correspond to the “3-residue insertion” (Fig. 1*B*). The absence of one leucine may reduce the hydrophobic interaction of the two molecules at this location, resulting in different turning points of the A and B chains (303EAE305 in chain A and 299ETF301 in chain B) (Fig. 1*C*).

### The unique LZ-Ext region is critical for USF2 function

**Figure 2:**
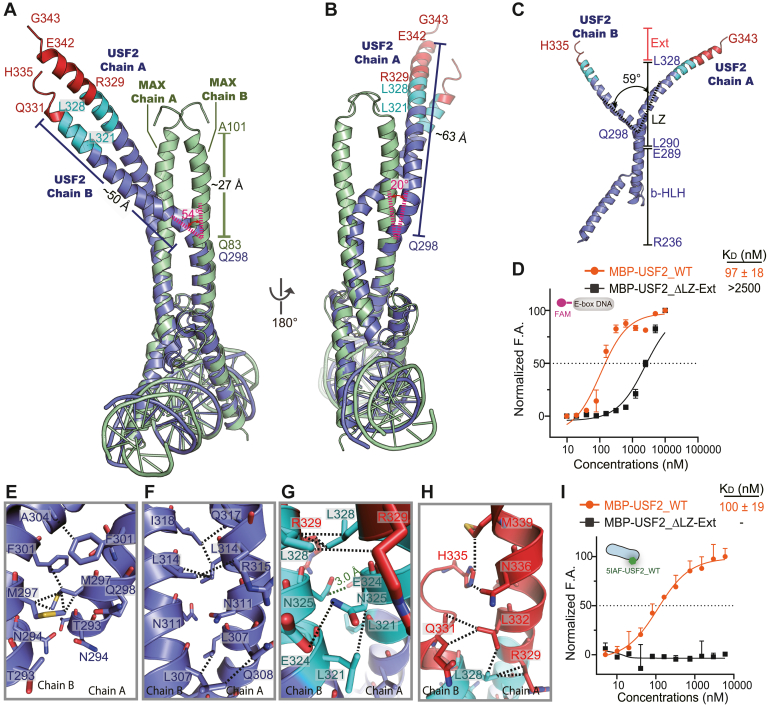
**The distinct bent and elongated LZ in USF2.***A* and *B*, superimposition of the USF2 structure (*purple*) and MAX structure (PDB code: 1AN2, *green*). The additional leucine zipper (Leu321–Leu328) and Ext region (Arg329–Gly343) are shown in *cyan* and *red*, respectively. The LZ-Ext region of USF2 above Gln298 is significantly different from that of MAX, with chain A having a 20° rotation and chain B having a 54° rotation. The LZ-Ext of USF2_chain A is ∼63 Å, which is more than twice as long as that of MAX (∼27 Å), and the LZ-Ext of USF2_chain B is ∼50 Å, which is approximately 23 Å longer than that of MAX. *C*, superposition of the two chains of the USF2 dimer shows that the LZ-Ext helices are asymmetric from the separation point Gln298 and twist approximately 59° from each other. *D*, serial dilutions of MBP-USF2_WT or MBP-USF2_△LZ-Ext were titrated to 20 nM FAM labeled E-box DNA, and the fluorescence anisotropy was measured. Error bars represent the SDs of four technical replicates. Removing LZ-Ext significantly reduced the DNA binding ability. *E*–*H*, residues on LZ-Ext from both chain A and chain B form hydrophobic interactions or H-bonds that facilitate the dimer formation of USF2. The H-bond is shown as *green dotted lines* and the distances are indicated, and other atomic contacts within 4 Å are shown as *black dotted lines*. *I*, fluorescence anisotropy assay to quantify the USF2 dimer formation. Serial dilutions (0–6000 nM) of MBP-USF2_WT or MBP-USF2_△LZ-Ext were titrated to 10 nM 5IAF labeled USF2_WT (_5IAF_USF2_WT), and the fluorescence anisotropy was measured. Error bars represent the SDs of four technical replicates. MBP-USF2_WT forms a dimer with a *K*_*d*_ of 100 ± 19 nM. MBP-USF2_△LZ-Ext had no detectable signal for dimerization. MAX, MYC-associated factor X; PDB, Protein Data Bank.

Superimposition of the complex structure of USF2-E box DNA with that of MAX-E box DNA shows that the bHLH structure of USF2 closely resembles that of the homologous protein MAX (Fig. 2*A*). Similarly, a previous study showed that the bHLH structure of USF1 also closely resembles that of MAX (20). However, the LZ-Ext region of USF2 above Gln298 is significantly different from that of MAX. Chain A has a 20° rotation, and chain B has a 54° rotation (Fig. 2, *A* and *B*). Superimposition of one subunit to the other in the USF2 dimer shows that the LZ-Ext helices are asymmetric and twist approximately 59° from each other (Fig. 2*C*).

Because of the additional leucine spaced seven residues (from Leu321 to Leu328) and the Ext region, the LZ-Ext helix of USF2 is significantly longer than that of other bHLH-LZ transcription factors. For example, the LZ-Ext of USF2_chain A is ∼63 Å, which is more than twice as long as that of MAX (∼27 Å), and the LZ-Ext of USF2_chain B is ∼50 Å, which is approximately 23 Å longer than that of MAX (Fig. 2, *A* and *B*). These results all show that USF2 has a distinct LZ-Ext region. Here, we also compared the structure of USF2 with MiT/TFE members, including TFE3 (Protein Data Bank [PDB] code: 7F09), TFEB (PDB code: 7Y62), MITF-apo (PDB code: 4ATH), and MITF-DNA (PDB code: 7D8T). The results show that although the USF2 and MiT/TFE members share a similar bent LZ structure, the length and degree of curvature of the LZ of USF2 are greater than those of the other members (Figs. S3–S6).

To study the role of LZ-Ext in interacting with E-box DNA, we designed an electrophoretic mobility shift assay (EMSA) using fluorophore Cy3 labeled E-box (_Cy3_E-box). The results showed that the bHLH-LZ-Ext of USF2 with or without MBP fusion formed complexes with the _Cy3_E-box in a dose-dependent manner (Fig. S7). USF2_ΔLZ-Ext (removing the LZ-Ext region) lost its interaction with the _Cy3_E-box (Fig. S7). In the fluorescence anisotropy (FA) assay with increased sensitivity compared with EMSA, the E-box binding ability of USF2_ΔLZ-Ext decreased by more than 20-fold compared with that of USF2_WT (Fig. 2*D*). Together, these results confirm that LZ-Ext is critical for USF2 to bind E-box DNA, consistent with a previous study (29).

Using CD analysis of USF2_ΔLZ-Ext and USF2_WT, we observed that the removal of the LZ-Ext region significantly reduced the secondary structure of USF2, with the percentage of alpha-helices decreasing from 42.5% to 11.2%, indicating that USF2_ΔLZ-Ext is not fully folded. Interestingly, adding E-box DNA increased the percentage of alpha-helices in USF2_ΔLZ-Ext from 11.2% to 28.5%, indicating that the E-box DNA can stabilize the USF2_ΔLZ-Ext and partially recover its disordered secondary structure. Of note, the percentage of alpha-helices in the USF2_WT and E-box DNA complex was 98%, showing that LZ-Ext was required to form a fully folded structure by interacting with E-box DNA (Fig. S8).

The key role of LZ-Ext in the binding of E-box DNA may be through the stabilization of the USF2 dimer. Extensive residues in LZ-Ext, including Met297, Phe301, Leu307, Leu314, Arg315, Leu321, Leu328, Leu332, His335, and Asn336 contribute to dimer formation (Fig. 2, *E*–*H*). To evaluate the role of LZ-Ext in USF2 dimerization, we first examined the ability of USF_bHLH-LZ_ to dimerize by FA assays using 5-iodoacetamido-fluorescein (5IAF)-labeled USF2_bHLH-LZ_. In this assay, we assume that the labeled and unlabeled USF2 homodimeric or heterodimeric molecules can recombine freely and have equal binding affinity. We observed that USF2 forms a dimer with an apparent dissociation constant (Kd^app^) of 100 ± 19 nM (Fig. 2*I*). USF2_ΔLZ-Ext completely lost its dimer-forming ability (Fig. 2*I*). Together, these analyses show that the LZ-Ext region is critical for the dimerization and function of USF2.

### Formation of the USF2 tetramer

**Figure 3:**
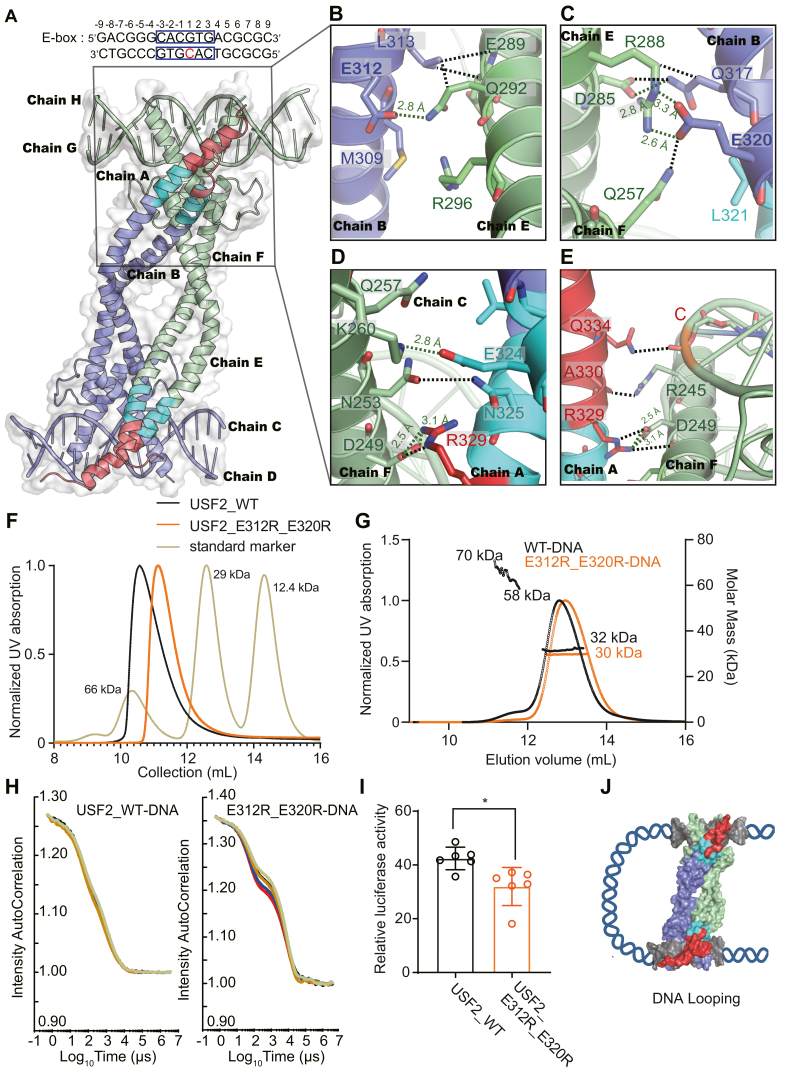
**USF2 tetramer formation.***A*, crystal structure of the USF2 tetramer. The additional leucine zipper (Leu321–Leu328) and Ext region (Arg329–Gly343) are shown in *cyan* and *red*, respectively. *B*–*E*, zoomed-in view of the hydrophobic interactions and salt bridges of residues between LZ-Ext of dimer AB and the bHLH region of dimer EF. The H-bonds are shown as *green dotted lines* and the distances are indicated, and other atomic contacts within 4 Å are shown as *black dotted lines*. *F*, size exclusion chromatography (SEC) assay shows that the retention time of the USF2_E312R_E320R double mutant is longer than that of the WT USF2_bHLH-LZ-Ext protein (denoted as USF2_WT). *G*, multiangle light scattering coupled with size-exclusion chromatography (SEC-MALS) analysis of USF2_WT and USF2_E312R_E320R in complex with E-box DNA. The differential refractometry index is shown along with the molecular mass of full complexes. Four percent of the WT complex was eluted as a tetramer. In contrast, the USF2_E312R_E320R-DNA complex was only eluted as dimers. *H*, dynamic light scattering (DLS) characterization of USF2_bHLH-LZ-ext-DNA complexes. *I*, luciferase reporter assay showing that the USF2_E312R_E320R mutant was defective in transcription in HeLa cells. Error bars represent the SDs of six independent biological replicates. *p* ∗ <0.05 by Student's unpaired *t* test. *J*, schematic illustration of a DNA-looping model using the surface of the USF2 tetramer structure. 5IAF, 5-iodoacetamido-fluorescein; LZ, leucine zipper; PDB, Protein Data Bank; USF, upstream stimulating factor.

As mentioned above, there are two dimeric USF2-DNA complexes in one ASU of the crystal. In fact, the two dimers further form a compact tetramer in rotational symmetry with a two-fold axis (Fig. 3*A*). The elongated LZ-Ext contributes extensively to tetramer formation. The residues Asn325 and Arg329 on chain A form H-bonds and an ionic bond with Asp249 and Asn253 on chain F. Glu312, Leu313, Gln317, Glu320, and Glu324 on chain B form an ionic bond, H-bonds and hydrophobic interactions with Asp285, Arg288, Glu289, and Gln292 on chain E and Gln257 and Lys260 on chain F (Fig. 3, *B*–*E*). Using proteins, interfaces, structures and assemblies (PISA) analysis, we calculated the interface area between the homodimers. The results showed that the buried area is approximately 1135 Å^2^, of which chain A contributes 43% and chain B contributes 57%. The dimer basic region contributes approximately 595 Å^2^, and the LZ region contributes approximately 540 Å^2^ (Fig. S9*A*). With this assembly, Gln334 in the Ext region forms an interaction with the phosphate backbone of the C(1) base in E-box DNA through the -NH_2_ group (Fig. 3, *A* and *E*). The FA assay result suggested that this interaction may contribute to the DNA binding ability of USF2 (Fig. S10).

Consistent with the contribution of Glu312 and Glu320 to the tetramer interaction, the retention time of USF2_E312R_E320R double mutant was longer than that of USF2_bHLH-LZ-Ext WT protein in the size-exclusion chromatography (SEC) (Fig. 3*F*). To rule out the effect of protein shape on retention time, we used SEC with multiangle light scattering (SEC-MALS) to determine the oligomerization state of USF2 in solution. Since the addition of E-box DNA increases the percentage of alpha-helices in USF2 to 98%, we performed SEC-MALS measurements using the USF2_bHLH-LZ-Ext-DNA complex. The results showed that the WT USF2-DNA complex was mainly eluted as a dimer, which contains one duplex DNA and has a theoretical molecular weight of 38 kDa, and 4% of the complex was eluted as a tetramer, which contains two duplex DNA and has a theoretical molecular weight of 76 kDa (Fig. 3*G*). In contrast, the tetramer interface mutant USF2_E312R_E320R was only eluted in a dimer peak (Fig. 3*G*). We further repeated the previously reported dynamic light scattering (DLS) experiment (20). Consistent with the previous report, the autocorrelation function of the WT protein showed a generally continuous decay rate state (Fig. 3*H*), with an estimated molecular weight of 74 kDa, consistent with a tetramer (Fig. S11*A*). In contrast, the autocorrelation function of the tetramer interface mutant showed two distinct decay rate states (Fig. 3*H*), suggesting that it contains two components. The estimated average molecular weight is 60 kDa (Fig. S11*B*), suggesting that the sample contained 75% tetramer and 25% dimer. These results supported that the E312R/E320R double mutation reduced the assembly of the tetramer. To study the influence of the USF2 tetramer on its transcriptional activity, we performed a luciferase reporting assay. The results showed that the transcriptional activity of the USF2_E312R_E320R mutant was significantly decreased compared with that of USF2_WT (Fig. 3*I*). Thus, tetramer formation is required for the adequate transcriptional function of USF2. These results provide the structural basis and support the previous speculation that the tetramerization of USFs exerts an effect on transcription through a DNA-looping mechanism (Fig. 3*J*).

### The interactions between the basic region of USF2 and E-box DNA

**Figure 4:**
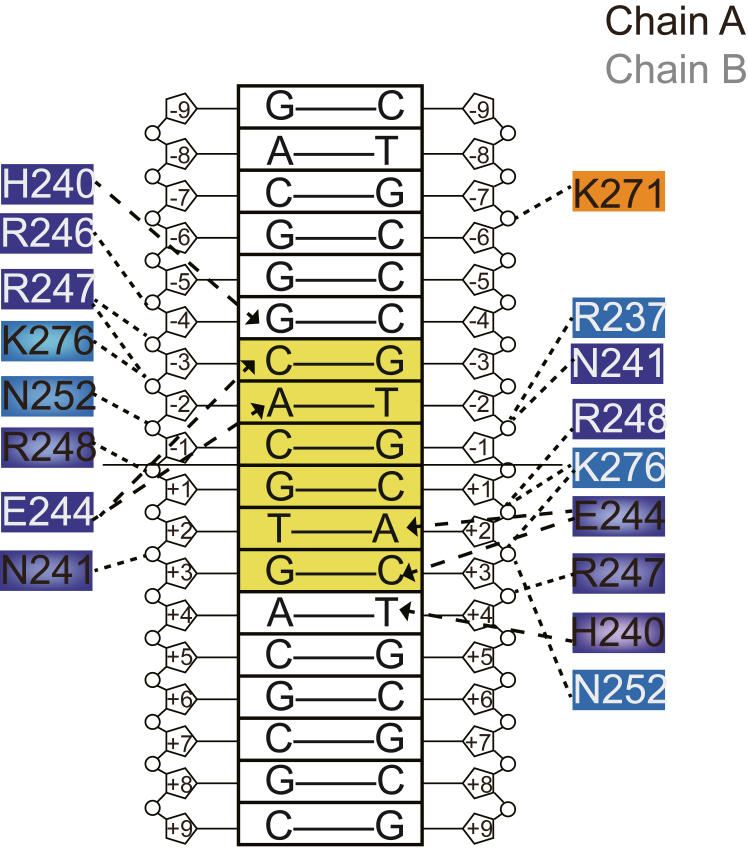
**Interactions between USF2 and E-box DNA.** Different background colors indicate that residues located in different regions, color coding: basic region-*blue*, Helix1 and Helix2 regions-marine and Loop region-*orange*. Hydrogen bonds are indicated with *dashed arrows*. Where *bold dashed arrows* indicate base-specific interactions, the remaining interactions are highlighted by *thin dashed lines*. The core motif of E-box DNA is highlighted in *yellow*. All the indicated hydrogen bonds are within a distance of 3.5 Å. USF, upstream stimulating factor.

The complex structure reveals how USF2 recognizes E-box DNA. The backbone of E-box DNA is extensively recognized by Arg237, Arg246, Arg247, Lys276, Asn252, Arg248, and Asn241. Glu244 recognizes the bases at positions 2, 3, −2, and −3. His240 recognizes the flanking bases at positions 4 and −4 (Fig. 4). The structure indicates that USF2 recognition of E-box DNA with the central palindromic sequence CACGTG is not completely symmetric (Fig. 4).

**Figure 5:**
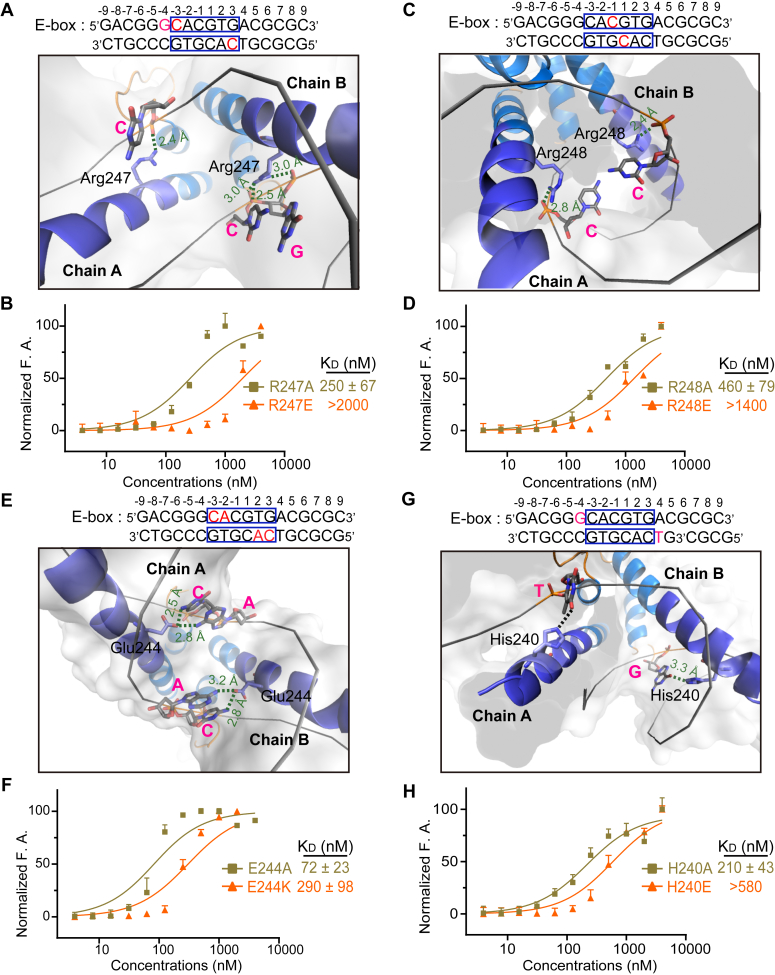
**The recognition of the backbone and base pairs of E-box DNA by the basic region of USF2**. *A*, residue Arg247 interacts with the backbone at the −3 and −4 positions of one strand and the −3 position of the other strand. *B*, fluorescence anisotropy shows that the R247A and R247E mutants interacted with E-box DNA with K_d_ values of 250 ± 67 nM and over 2000 nM, respectively. Error bars represent the SDs of four technical replicates. *C*, residue Arg248 produces a symmetric interaction with the backbone at the −1 position of both E-box DNA strands. *D*, according to the fluorescence anisotropy assay, the R248A and R248E mutants interacted with E-box DNA with *K*_*d*_ values of 460 ± 79 nM and over 1400 nM, respectively. Error bars represent the SDs of four technical replicates. *E*, residue Glu244 provides base-specific interactions with two base pairs at the −2 and −3 positions of the different strands. *F*, according to the fluorescence anisotropy assay, the E244A and E244K mutants interacted with E-box DNA with *K*_*d*_ values of 72 ± 23 nM and 290 ± 98 nM, respectively. Error bars represent the SDs of four technical replicates. *G*, residue His240 provides base-specific interactions with the base pairs at the −4 position of the different strands. *H*, the H240A and H240E mutants interacted with E-box DNA with *K*_*d*_ values of 210 ± 43 nM and over 580 nM, respectively. Error bars represent the SDs of four technical replicates. For (*A*, *C*, *E*, and *G*), the H-bonds are shown as *green dotted lines* and the distances are indicated, and other atomic contacts within 4 Å are shown as *black dotted lines*. USF, upstream stimulating factor.

Next, we designed a series of USF mutations in the basic region to evaluate their contributions to E-box DNA recognition. According to the structure, Arg247 binds the backbone of DNA at positions −4 and −3. Diminishing these salt bridge interactions by the R247A mutation slightly decreased the K_d_ to 250 ± 67 nM, while introducing the repulsive mutation R247E reduced the interaction by more than 20-fold (Fig. 5, *A* and *B*). Similarly, the R248E mutation reduced the interaction between USF2 and E-box DNA by more than 14-fold (Fig. 5, *C* and *D*).

Glu244 in the basic region of USF2 recognizes the A(2) and C(3) bases of the E-box in a symmetric manner (Fig. 5*E*). Interestingly, we observed that E244A/K mutations only slightly affected the DNA binding ability of USF2, with K_d_ values of 72 ± 23 nM and 290 ± 98 nM, respectively (Fig. 5*F*), suggesting that this residue mainly serves to recognize specific sequences rather than provide strong binding affinity. In contrast, His240 contributed significantly to the binding affinity. The H240A mutation decreased the K_d_ to 210 ± 43 nM, and the repulsive mutation H240E reduced the interaction by more than 5-fold (Fig. 5, *G* and *H*).

**Figure 6:**
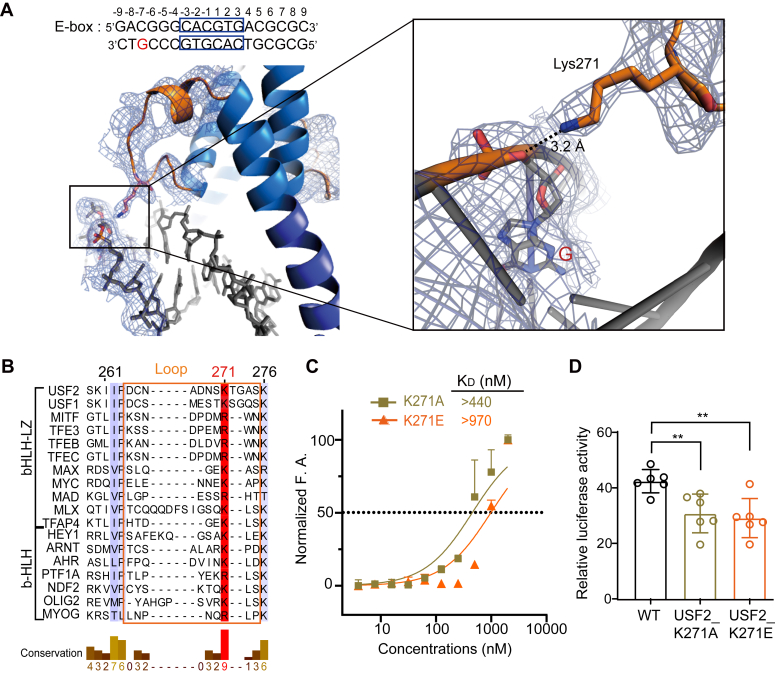
**Conserved Lys271 in the loop region of USF2 participates in E-box DNA interactions.***A*, Lys271 in the loop region interacts with the backbone of G(−7). *B*, sequence alignment between the bHLH and bHLH-LZ transcription factors in the loop region. All 18 analyzed proteins present a conserved basic amino acid in the loop region. *C*, fluorescence anisotropy showing that the K271A mutation decreased the DNA binding ability with a K_d_ over 440 nM, and the repulsive mutation K271E significantly decreased the interaction with a *K*_*d*_ over 970 nM. Error bars represent the SDs of four technical replicates. *D*, luciferase reporter assay showing that USF2_K271A and USF2_K271E mutants significantly reduced transcriptional activity compared with USF2_WT in HeLa cells. Error bars represent the SDs of six independent biological replicates. *p* ∗∗ <0.01 by Student's unpaired *t* test. USF, upstream stimulating factor.

Finally, we observed that Lys271 in the loop region binds the backbone of G(−7) through a salt bridge (Fig. 6*A*). Interestingly, although the loop sequence of the bHLH-LZ transcription factors is highly variable, the residues at position 271 are more conserved, usually arginine or lysine, in eighteen analyzed bHLH-LZ and bHLH transcription factors (Fig. 6*B*). We also performed an FA assay to assess whether this conserved basic residue participates in E-box DNA binding. The results showed that the K271A mutation decreased the DNA binding ability with a *K*_*d*_ over 440 nM (Fig. 6*C*). The repulsive mutation K271E decreased the interaction by more than 9-fold (Fig. 6*C*). Consistently, the two mutations significantly reduced the transcriptional activity compared with the WT protein according to the luciferase reporting assay (Fig. 6*D*). These results confirmed that this conserved Lys271 on the loop is critical in the E-box DNA interaction.

## Discussion

Here, we report the complex structure of USF2 and E-box DNA, revealing the role of the bent elongated LZ region in tetramer formation and transcription activity and the molecular mechanism by which USF2 recognizes E-box DNA.

**Figure 7:**
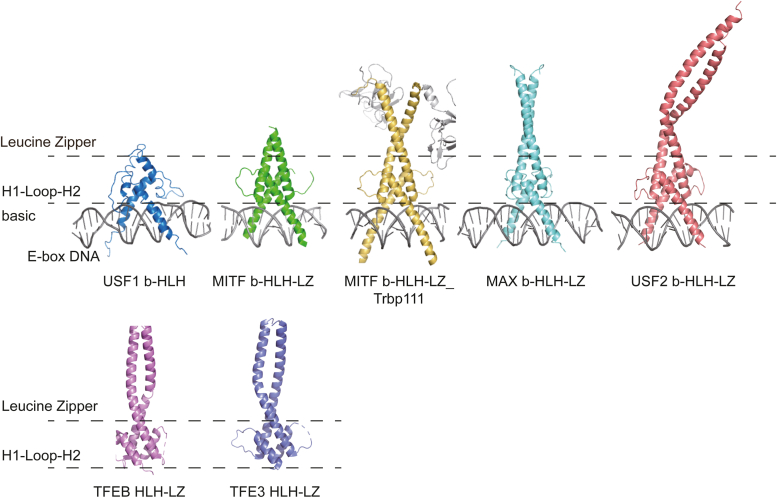
**Structural comparison of USF1/2, MAX/MYC and MiT/TFE family members.** From *left* to *right*: USF1 (bHLH-DNA, PDB code: 1AN4), MITF (bHLH-LZ-DNA, PDB code: 4ATK), MITF (bHLH-LZ-Trbp111- DNA, PDB code: 7D8T), MAX (bHLH-LZ-DNA, PDB code: 1AN2), USF2 (bHLH-LZ-DNA, PDB code: 8IA3), TFEB (HLH-LZ, PDB code: 7Y62), TFE3 (HLH-LZ, PDB code: 7F09). MAX, MYC-associated factor X; MITF, microphthalmia-associated transcription factor; PDB, Protein Data Bank; TFEB, transcription factor EB.

USF2 contains a “3-residue insertion” in the LZ region that characterizes the MiT/TFE/USF subfamily and distinguishes it from the MYC/MAX/MAX dimerization protein subfamily. Previous studies have shown that this unique “3-residue insertion” of MITF is critical for its selective dimerization within MiT/TFE subfamily members, as removing the insertion allows its dimerization with MAX (26, 30). This unique insertion results in the off-registered LZ and may further lead to the dynamic properties of the LZ region. Consequently, in the complex structures of MITF-E-box DNA and MITF-M-box DNA, the LZ region of MITF is untraceable (26). It can only be resolved in crystal structures with the aid of the fusion protein Trbp111 (PDB: 7D8T) or in the absence of DNA (PDB: 4ATH). The structure of USF2 reported here reveals the first full picture of the bHLH-LZ domain of the MiT/TFEB/USF subfamily in the presence of E-box DNA without the aid of a fusion protein (Fig. 7). The elongated LZ-Ext might help to stabilize the dynamic LZ and further allow the resolution of the long and bent structure.

USF1 was reported to form a tetramer in solution according to previous studies (20, 31). Here, we observed that USF2 also formed a tetramer according to the crystal structure and DLS experiments (Fig. 3, *A* and *H*). We analyzed the residues involved in the formation of USF2 tetramers by ConSurf and sequence alignment and found that a subset of these residues is highly conserved in USF1 and USF2. These results indicate that USF1 and USF2 may form tetramers based on a conserved molecular mechanism (Fig. S9, *B* and *C*).

Notably, in the SEC-MALS experiment, we observed that only 4% of the USF2-DNA complex was tetrameric (Fig. 3*G*). The tetramer and dimer concentrations were roughly estimated to be 0.35 μM and 16.8 μM, respectively. This resulted in an estimated tetramer dissociation constant of about 800 μM. This is inconsistent with the DLS results, because when we diluted the WT protein to a concentration containing approximately 25 μM tetramers, the autocorrelation function remained similar to that of the high concentration, suggesting that the tetramer dissociation constant is possibly in the low micromolar range (Fig. S11*C*). Although the molecular sieve effect driving the equilibrium shift in the dissociative direction may have caused the tetramer affinity to be underestimated, we should remain open to whether the tetramer observed in the crystal structure is an artifact. In the future, it would be valuable to test more closely with physiological conditions.

We also observed that the highly conserved Lys271 of USF2 participates in DNA interactions. Interestingly, our recent work shows that the Food and Drug Administration-approved drug molecule eltrombopag binds this semiconservative site in TFEB (Arg271 in the loop region) and disrupts the interaction between TFEB and the CLEAR DNA element (27). These results indicate that the basic amino acid on the loop region may present a common DNA binding and a potential targeting pocket of the bHLH and bHLH-LZ transcription factors for drug design.

Finally, the tetramer of USF2 *via* the elongated and bent LZ-Ext provides the molecular structural basis of USF2-DNA looping, which can help to understand the role of USF2 as one of the key components of the transcription machinery.

## Experimental procedures

### Cloning and expression

Full-length human USF2 isoform one was amplified from the complementary DNA of HeLa cells using polymerase chain reaction and inserted into a pET-22b (+) vector. To purify MBP-fused USF2 proteins, the bHLH-LZ-Ext domain (also denoted as USF2_WT, residues 235–346) and the bHLH domains (also denoted as USF2_ΔLZ-Ext, residues 235–292) were constructed in a pHis-MBP-TEV vector to generate MBP-USF2_WT and MBP-USF2_ΔLZ-Ext. The plasmid was introduced into *Escherichia coli* (*E. coli*) strain Rosetta (DE3) competent cells, and the cells were grown in Luria broth medium containing 50 μg·ml^−1^ kanamycin at 37 °C. When the absorbance at 600 nm reached 0.6 to 0.8, the cell culture was cooled to 16 °C, and the protein expression was induced by adding 0.5 mM IPTG into the culture. After 20 h of induction at 16 °C, the cells were harvested by centrifugation and purified to homogeneity with a Ni-HiTrap affinity column and a size exclusion Superdex 75 column (GE HealthCare). MBP-CGG-USF2_WT and other MBP-USF2 mutants including H240A, H240E, E244A, E244K, R247A, R247E, R248A, R248E, K271A, K271E, and E312R_E320R, were all generated based on the construct MBP-USF2_WT and purified according to the procedure mentioned above.

To obtain USF2_WT, USF2_ΔLZ-Ext, and CGG-USF2_WT, MBP-USF2_WT, MBP-USF2_ΔLZ-Ext, and MBP-CGG-USF2_WT were first cleaved by tobacco etch virus protease to remove the His-MBP tag. The cleaved protein was then passed through a Ni-HiTrap column or a HiTrap MBP column followed by purification with a HiTrap SP Sepharose column and a size exclusion S75 column (GE HealthCare). For preparation of the 5-IAF-labeled protein USF2_WT (denoted as _5IAF_USF2_WT), the sulfhydryl cysteine end of CGG-USF2_WT was labeled with Thermo Fisher Scientific 5-IAF per the manufacturer's instructions.

### Crystallization, data collection, and structure refinement

The USF2_bHLH-LZ-Ext protein was concentrated to 20 mg ml^−1^ in Superdex 75 running buffer by centrifugal ultrafiltration (Millipore, 3 kDa cutoff) before crystallization trials. The protein was mixed with 18 bp double-stranded E-box DNA from human *HOXA9* containing a single nucleotide overhang at each 3′ end (5′-GACGGGCACGTGACGCGC-3′, 5′-CTGCCCGTGCACTGCGCG-3′). Initial crystallization trials were carried out using the sitting-drop vapor-diffusion method at 18 °C with a series of commercial crystal screen kits including Morpheus, SG1 Screen, BCS Screen, ProPlex, LFS, JCSG Plus, Ligand-Friendly Screen, PACT Premier, Macrosol, and Stura FootPrint Screens from Molecular Dimensions. Each drop was prepared by mixing 0.5 μl protein solution containing 23 mg ml^−1^ protein with 0.5 μl reservoir solution and was equilibrated against 60 μl reservoir solution. The initial crystals appeared after 5 days under the following condition: 0.1 M Tris pH 7.0, 20% PEG4000 (from the Proplex Screen). The concentration of the salt and the precipitant was adjusted in further optimization of the crystallization conditions using the sitting-drop method in 96-well plates at 18 °C. After optimization, crystals of high quality were obtained for data collection. The optimized bHLH-LZ crystal mounted in a loop was soaked briefly in a cryoprotectant solution consisting of the corresponding reservoir solution supplemented with 20% (v/v) glycerol and was then flash-frozen in liquid nitrogen. X-ray diffraction data were collected on a beamline 19U at the Shanghai Synchrotron Radiation Facility using an EIGER 16M detector (32). All frames were collected at 100 K using a 0.5 oscillation angle with an exposure time of 0.3 s per frame. The crystal-to-detector distance was set to 350 mm. The images were processed with the XDS package using the XDSgui interface (https://xds.mr.mpg.de/) (33). The structure was solved by molecular replacement using the program Molrep (34). The structure model of the USF2_bHLH-LZ domain predicted by Alphafold (35) and the E-box DNA model from PDB ID: 1AN4 were used as searching models in the molecular replacement process. Iterative model building and refinement were performed using Coot (https://www2.mrc-lmb.cam.ac.uk/personal/pemsley/coot/) and Phenix (https://phenix-online.org/) (36, 37). Data collection and refinement statistics are given in Table 1.

### Electrophoretic mobility shift assay

Duplex E-box DNA (5′- GAC GGG CACGTG ACG CGC -3′) was labeled with fluorophore Cy3 at the 5′ end in one chain. Serial dilutions of 6×HisMBP-USF2_WT (0–4 μM) or 500 nM MBP control were incubated with 100 nM _Cy3_E-box in buffer containing 10 mM Tris pH 8.0, 50 mM KCl, 1 mM DTT, 2 mM MgCl_2,_ and 5% (v/v) glycerol at 4 °C for 2 h. The resulting DNA–protein complex and free DNA were separated by a 6% nondenaturing polyacrylamide gel. The gel was photographed with an Amersham Imager 600 (GE HealthCare) in fluorescence detection mode with stimulation light at 520 nm and a Cy3 filter.

### FA assay

FA measurements were carried out using a SpectraMax M5 microplate reader (Molecular Devices) and TECAN M1000 PRO. To determine the DNA binding activity of MBP-USF2_WT and MBP-USF2_△LZ-Ext, FAM-labeled E-box DNA (5′-FAM-GACGGGCACGTGACGCGC-3′, and the complementary DNA sequence was 5′-CTGCCCGTGCACTGCGCG-3′, final concentration 20 nM) was mixed with a gradient of USF2_WT or MBP-USF2_△LZ-Ext (0–10,000 nM) in buffer containing 20 mM Tris pH 8.0, 50 mM KCl, 2 mM MgCl_2,_ and 0.5% glycerol. The mixtures were incubated at 4 °C for 3 h before measurement. An excitation beam at 480 nm and emission at 535 nm were used to read the FA signal.

To determine the dimer formation of USF2, _5IAF_USF2_WT was mixed with a gradient of MBP-USF2_WT or MBP-USF2_△LZ-Ext at the indicated concentrations (0–6 μM) in buffer containing 20 mM Tris 8.0, 50 mM KCl, 2 mM MgCl_2_, and 0.5% glycerol. The mixtures were incubated at 4 °C overnight before measurement. An excitation beam at 494 nm and emission at 518 nm were used to read the FA signal.

The binding affinity Kd was calculated using the equation with receptor depletion:

$$A=A_{f}{+(A}_{b}-A_{f})\times \frac{\left(L_{T}+K_{d}+R_{T}\right)-\sqrt{{\left(L_{T}+K_{d}+R_{T}\right)}^{2}-4L_{T}R_{T}}}{2L_{T}}$$

L_T_ = the total added concentration of ligand; A = the experimental anisotropy; A_f_ = the anisotropy for the free ligand; A_b_ = the anisotropy for the fully bound ligand.

### SEC assay

The protein was loaded onto a Superdex 75 Increase gel-filtration column (GE HealthCare, 10/300 GL) in buffer containing 20 mM Hepes pH 7.5 and 250 mM NaCl. The resulting data were exported and plotted using GraphPad Prism.

### SEC with multiangle light scattering

The USF2_bHLH-LZ protein (WT or E312R_E320R) at a concentrations of 700 μM was mixed with E-box DNA (350 μM) (so that the ratio between the USF2 dimer and duplex DNA is 1:1) and incubated overnight at 4 °C. The Superdex 75 Increase column was equilibrated with a buffer containing 20 mM Hepes pH 7.5, 100 mM KCl, and 10 mM DTT, and the flow rate was 0.5 ml/min. The light scattering signals were monitored on a Dawn HELEOS-II detector, concentrations of apo-protein samples were measured using an Optilab T-rEX refractive index detector (Wyatt Technology), and concentrations of protein–DNA complexes were measured using both refractive index and UV absorption readings. The molecular mass was calculated using ASTRA VI software (https://www.wyatt.com/products/software/astra.html) (Wyatt Technology).

### CD spectroscopy

CD spectra were obtained with an Applied Photophysics Chirascan spectropolarimeter at room temperature, using 0.05 cm path-length and 1 nm bandwidth, 1 s averaging time per point, measurements every 1 nm. The wavelength range measured by CD spectra is 180 to 260 nm. Average, blanked, and smoothed using software provided by the manufacturer. Blanks were taken before each data collection and subtracted from each other to monitor instrument drift. All spectra were collected with protein or protein-DNA complexes in CD buffer containing 20 mM Hepes and 10 mM KCl, pH 7.5. USF2_bHLH and USF2_bHLH-LZ were incubated with or without E-box DNA at a 1:2 M ratio for 3 h before SEC to remove the unbound E-box DNA and to change to CD buffer as mentioned above. The collected sample at a concentration of 20 μM was subjected to the following CD spectra and analyzed using the program CDNN. Due to the high background provided by 10 mM potassium chloride, the wavelength range of 195 to 260 nm with a lower background was selected for data processing.

### Dynamic light scattering

DLS was performed with a WYATT DynaPro detector. Molecular masses and hydrodynamic radii of gyration were calculated from the autocorrelation function using the manufacturer's software. Sample were taken into 100 mM KCl, 10 mM Hepes pH 7.5 by ultrafiltration and filtered through 0.02 μm filters to remove any dust particles. A sample concentration of 400 μM was selected according to the previous experimental conditions to ensure that the dynamic light scattering detector can detect a stable laser signal. Five sets of replicates were performed on each sample, ten independent measurements were taken for each set of experiments, and the reported values were calculated arithmetic means.

### Luciferase reporter assay

The whole genomic DNA of HEK 293T cells was extracted using the TIANamp Genomic DNA kit (Tiangen Biotechnology). The upstream transcription start site (−1/−2700 bp) of USF2 downstream of *HOXA9* was amplified. The amplified sequence was constructed on the PGL4.10 vector and denoted as PGL4.1/HOXA9. Full-length human USF2 isoform 1 and the mentioned mutants were constructed in the pcDNA3.1 vector. The PGL4.1/HOXA9 and PRL-TK vectors were cotransfected into HeLa cells (from American Type Culture Collection) with the USF2_WT and mutant plasmids, respectively. Tested with MycoBlue *Mycoplasma* Detector D101 (Vazyme), the cells were free from *mycoplasma* contamination. After 48 h, the cells were lysed and detected by a DL101-01 dual luciferase reporter kit (Vazyme).

## Data availability

Atomic coordinates and structure factors for the reported crystal structure have been deposited with the Protein Data Bank under the accession number 8IA3.

## Supporting information

This article contains supporting information.

## Conflict of interest

The authors declare that they have no conflicts of interest with the contents of this article.
