# Supplementary material for: Tetramerization of upstream stimulating factor USF2 requires the elongated bent leucine zipper of the bHLH-LZ domain
Source: J Biol Chem. 2023 Sep 9;299(10):105240. doi: 10.1016/j.jbc.2023.105240 (PMC10570711; doi:10.1016/j.jbc.2023.105240)
Supplement: Supporting Figures [file mmc1.pdf]

Supplementary Data

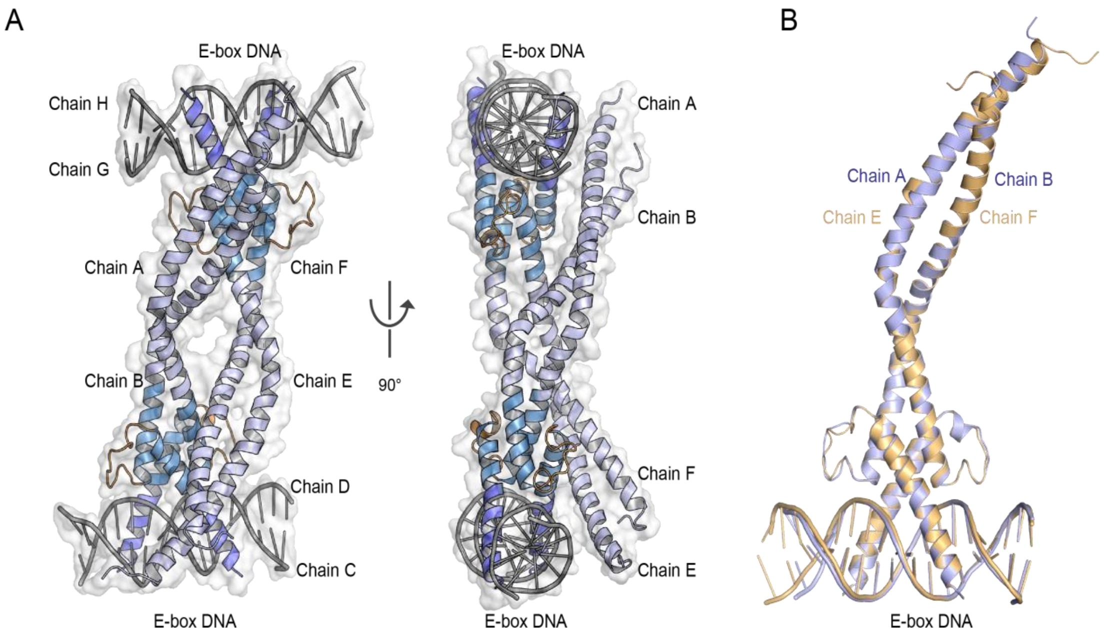

Figure S1. Two dimeric USF2-DNA complexes in one asymmetric unit of the crystal.

(A) One asymmetric unit (ASU) contains four USF2 protein chains (A, B, E, F) and four single strands of DNA (chains C, D, G, H).

(B) The structures of the two dimers closely resemble each other with a root-mean-square deviation (RMSD) of 0.36 Å.

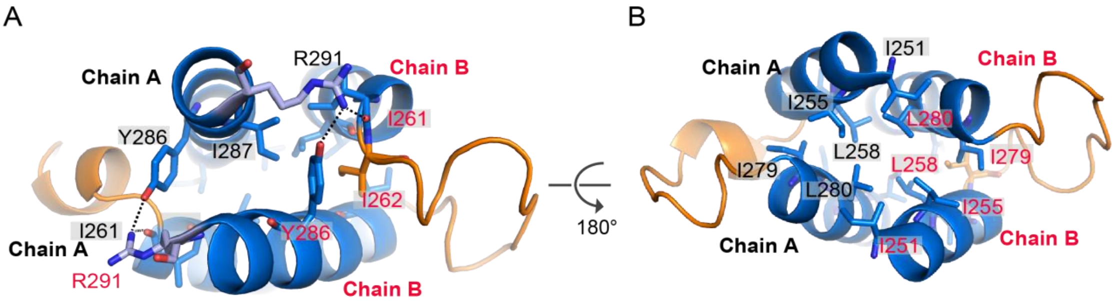

Figure S2. The zoomed-in view of the HLH region. Helix 1 (from Ile251 to Ile262) and helix 2 (from Lys276 to Glu289) of both chains form a hydrophobic four-helix bundle through interactions between residues Ile251, Ile255, Leu258, Ile261, Leu280, Tyr286 and Arg291.

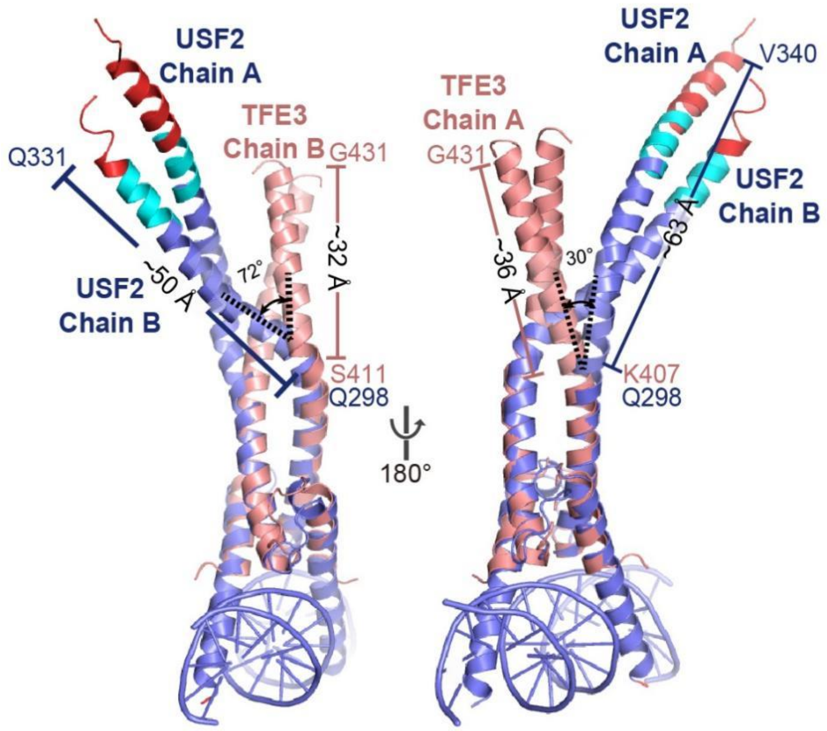

Figure S3. Superimposition of the USF2 structure and TFE3 structure (PDB code: 7F09). The additional leucine zipper (Leu321–Leu328) and Ext region (Arg329–Gly343) of USF2 are shown in cyan and red, respectively. The LZ-Ext region of USF2 is significantly different from that of TFE3, with chain A having a 30° rotation and chain B having a 72° rotation.

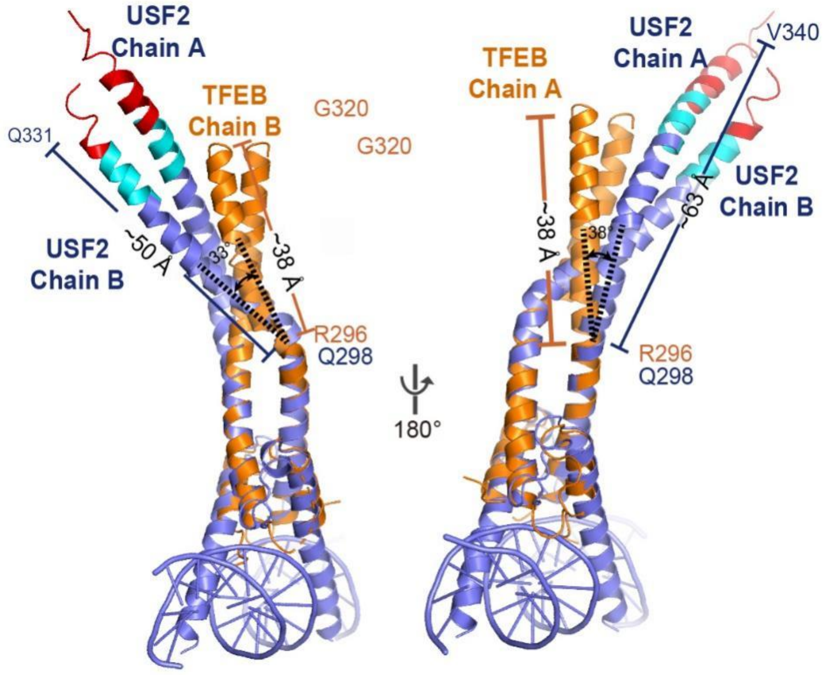

Figure S4. Superimposition of the USF2 structure and TFEB structure (PDB code: 7Y62). The additional leucine zipper (Leu321 – Leu328) and Ext region (Arg329 – Gly343) of USF2 are shown in cyan and red, respectively. The LZ-Ext region of USF2 is significantly different from that of TFEB, with chain A having a 38° rotation and chain B having a 33° rotation.

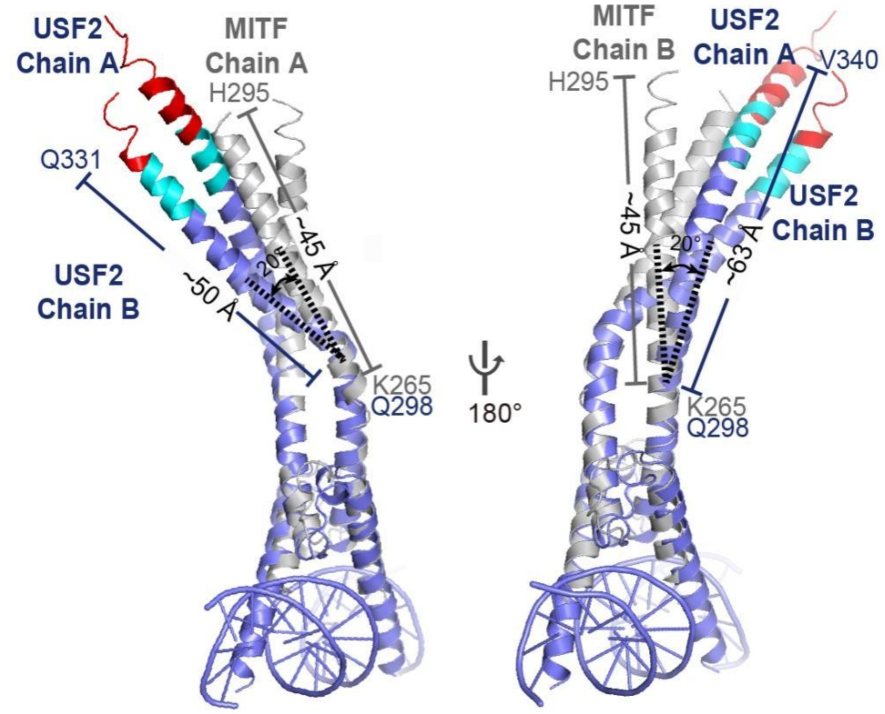

Figure S5. Superimposition of the USF2 structure and MITF apo structure (PDB code: 4ATH). The additional leucine zipper (Leu321 – Leu328) and Ext region (Arg329 – Gly343) of USF2 are shown in cyan and red, respectively. The LZ-Ext region of USF2 is significantly different from that of MITF, with chain A having a 20° rotation and chain B having a 20° rotation.

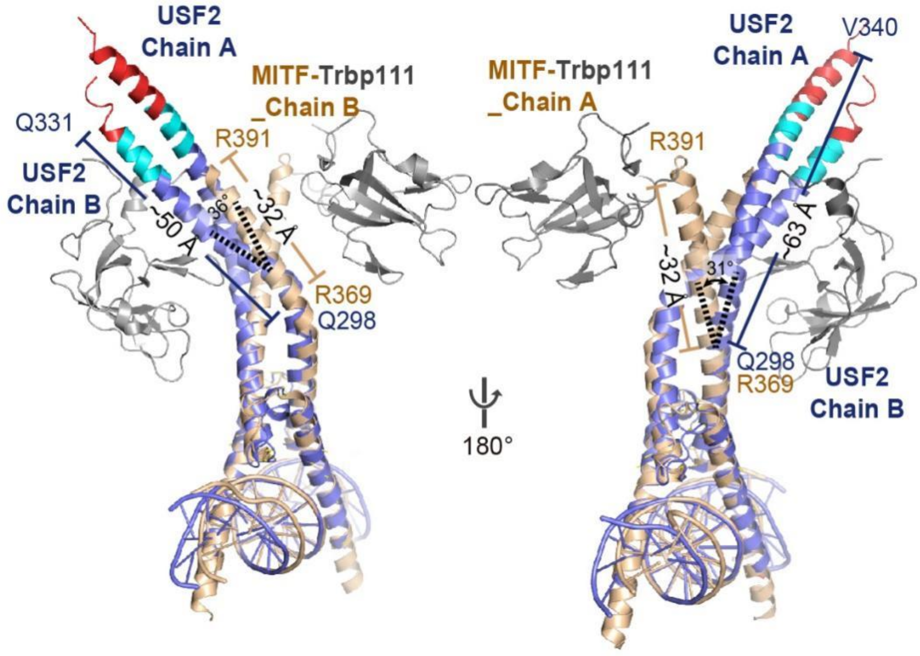

Figure S6. Superimposition of the USF2 structure and MITF-DNA complex structure (PDB code: 7D8T). The additional leucine zipper (Leu321 – Leu328) and Ext region (Arg329 – Gly343) of USF2 are shown in cyan and red, respectively. The LZ-Ext region of USF2 is significantly different from that of MITF, with chain A having a 31° rotation and chain B having a 36° rotation.

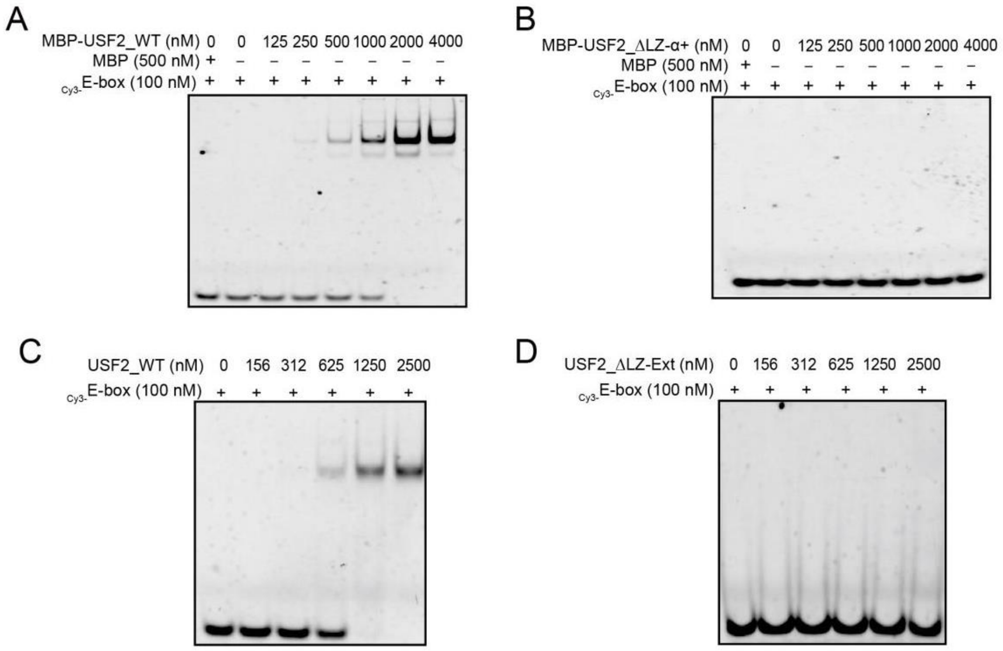

Figure S7. Electrophoretic mobility shift assay (EMSA) using a Cy3-labeled E-box ($_{Cy3}$E-box).

(A–B) MBP-USF2_WT formed complexes with the $_{Cy3}$E-box in a dose-dependent manner, and MBP-USF2_ΔLZ-Ext (removing the LZ-Ext region) lost its interaction with the $_{Cy3}$E-box.

(C–D) USF2_WT formed complexes with the $_{Cy3}$E-box in a dose-dependent manner, and USF2_ΔLZ-Ext (removing the LZ-Ext region) lost its interaction with the $_{Cy3}$E-box.

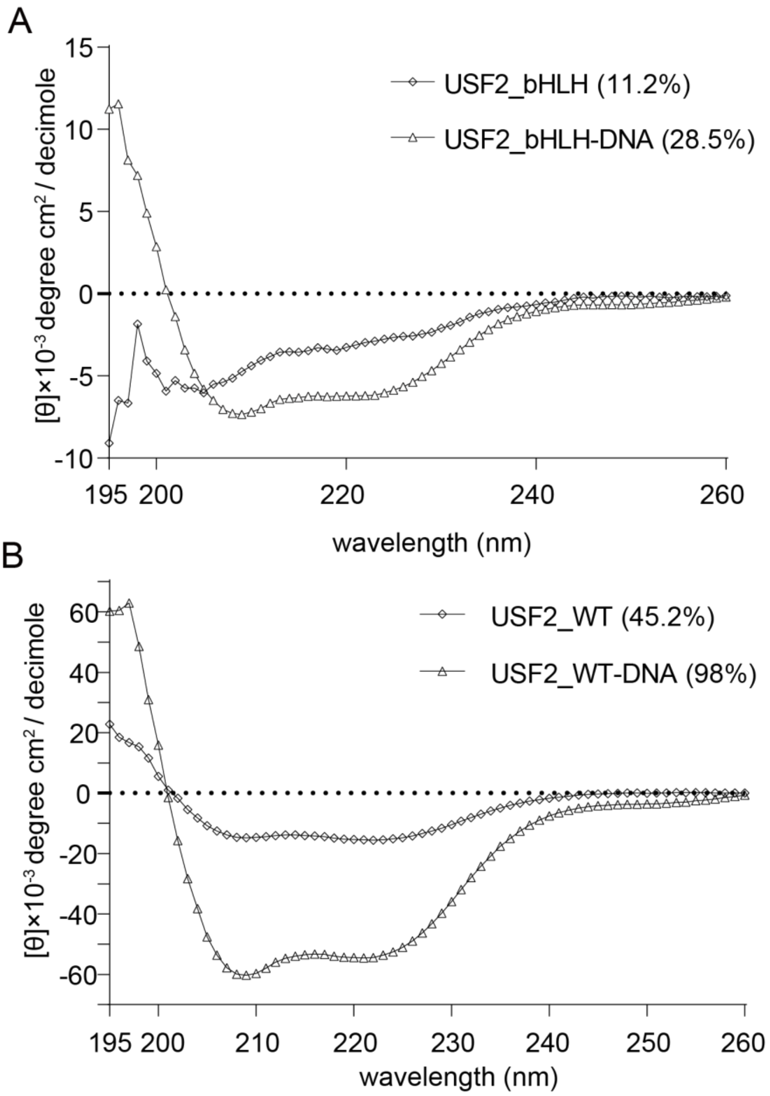

Figure S8. Normalized CD spectra.

(A) Spectra of free USF2_bHLH and a 1:2 molar mixture of USF2_bHLH with E-box DNA (USF2_bHLH-DNA). The estimated α-helical contents of USF2_bHLH and USF2_bHLH-DNA are indicated in parentheses.

(B) Spectra of free USF2_bHLHLZ (USF2_WT) and a 1:2 molar mixture of USF2_bHLHLZ with E-box DNA (USF2_WT-DNA). The estimated α-helical contents of USF2_WT and USF2_WT-DNA are indicated in parentheses.

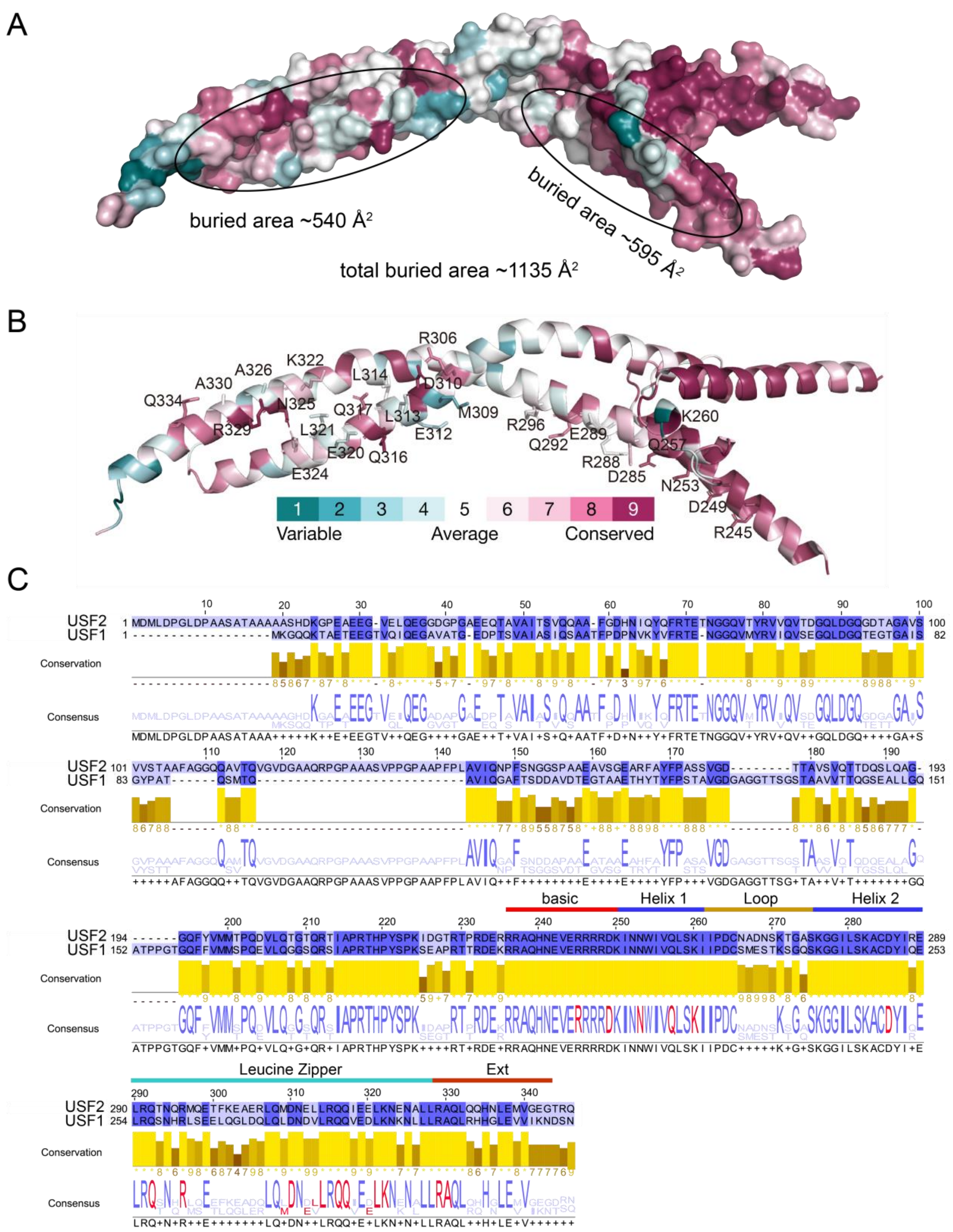

Figure S9. Residue conservation analysis of the USF family.

(A) The contact area of the tetramer is approximately 1135 Å^2. The dimer basic region contributes approximately 595 Å^2, and the LZ region contributes approximately 540 Å^2. Chain A contributes 43%, and chain B contributes 57%.

(B) A total of 111 sequences of USF1 and USF2 from different species were aligned and analyzed with ConSurf. The conservation level was represented using a color scale. Residues contributing to the tetramerization of USF2 are shown as sticks in the structure

of the USF2 dimer.

(C) Alignment of the full length sequences of USF1 and USF2. Residues contributing to the tetramerization of USF2 are marked in red.

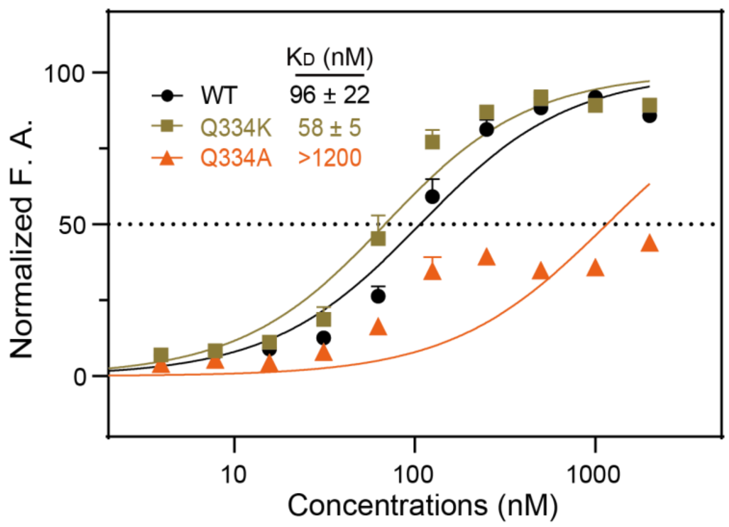

Figure S10. Fluorescence anisotropy assay detecting the interaction between FAME-box DNA and USF2_WT/Q334A/Q334K. The ability of USF2_Q334A mutant to bind E-box DNA was decreased, while the USF2_Q334K mutant interacted with E-box DNA similarly to USF2_WT. Error bars represent the SDs of four technical repeats.

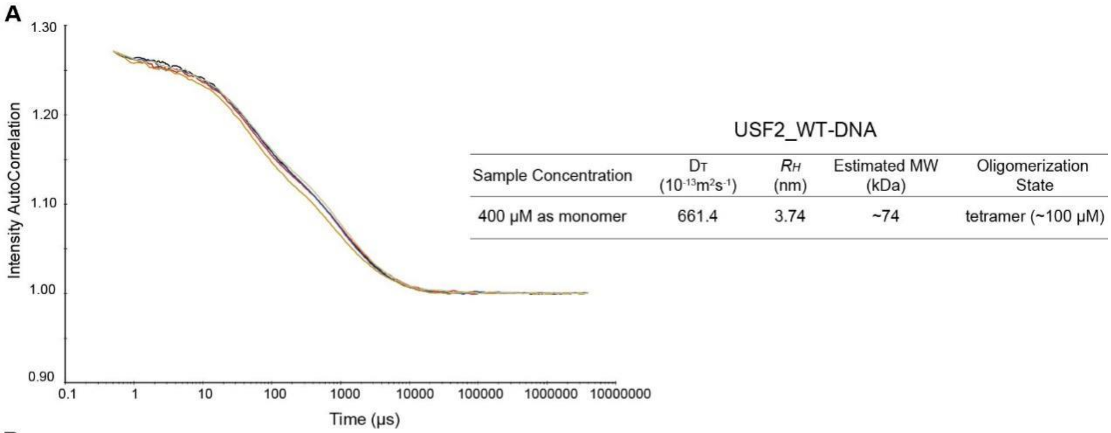

USF2_WT-DNA

Sample Concentration	DT ($10^{-13}m^2s^{-1}$)	*RH* (nm)	Estimated MW (kDa)	Oligomerization State
400 μM as monomer	661.4	3.74	~74	tetramer (~100 μM)

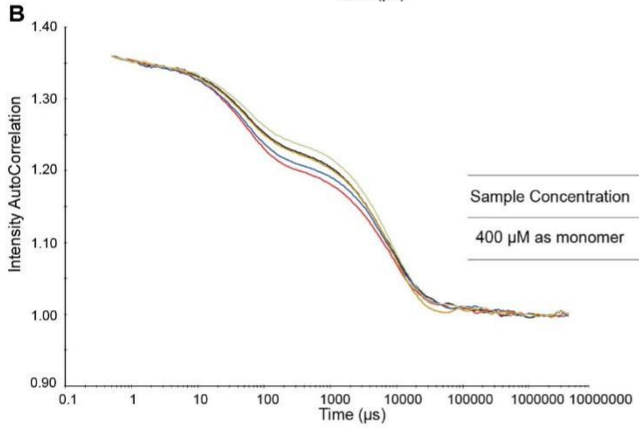

USF2_E312R_E320R-DNA

Sample Concentration	DT ($10^{-13}m^2s^{-1}$)	*RH* (nm)	Estimated MW (kDa)	Oligomerization State
400 μM as monomer	756.6	3.36	~60	tetramer (~75 μM)<br>dimer (~50 μM)

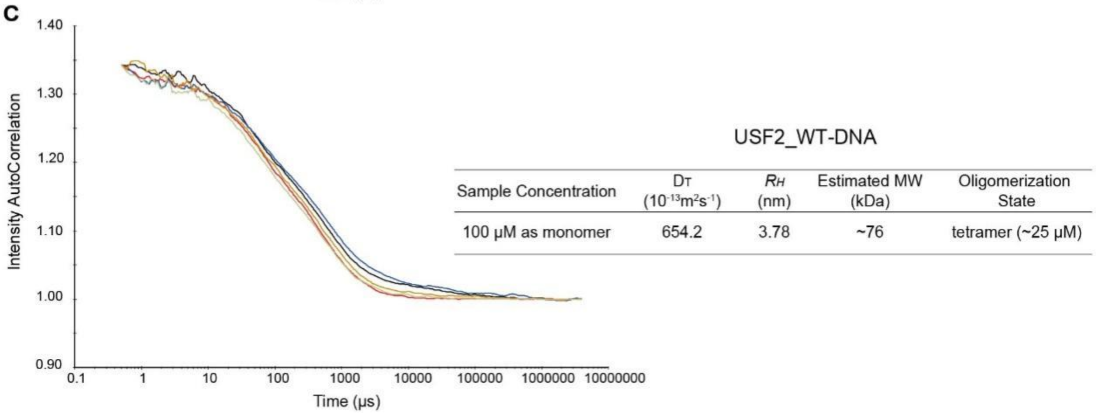

USF2_WT-DNA

Sample Concentration	DT ($10^{-13}m^2s^{-1}$)	*RH* (nm)	Estimated MW (kDa)	Oligomerization State
100 μM as monomer	654.2	3.78	~76	tetramer (~25 μM)

Figure S11. Dynamic light scattering (DLS) characterization of USF2_bHLH-LZ-ext-DNA complexes. Average of the translational diffusion coefficient (DT), hydrodynamic radius of rotation (*RH*), and estimated molecular weight of wild-type and double-mutant proteins with DNA complexes as determined by DLS. The tetramer contains two duplex DNA and has a theoretical molecular weight of 76 kDa. The dimer contains one duplex DNA and has a theoretical molecular weight of 38 kDa.

(A) The autocorrelation function of WT protein shows a generally continuous decay rate states.

(B) The autocorrelation function of E312R_E320R double mutant shows two distinct decay rate states, suggesting that it contains two components. Because the size of dimer and tetramer is difficult to separate, the estimated molecular weight is an average of the two oligomerization states. Based on this average molecular weight, we estimate that 75% of the USF forms tetramers. Accordingly, we estimate that the KD of the

tetramerization of the mutant is about 30 μM.

(C) Dilution of WT protein into ~25 μM tetramer did not change the shape of the autocorrelation function, suggesting that the K_D of the tetramerization of the WT protein is low.

For **(A-C)**, curves of 5 repeated samples were shown. Each curve is the average of 10 measurements.
